# Associations of blood glucose and prevalent diabetes with risk of cardiovascular disease in 500 000 adult Chinese: the China Kadoorie Biobank

**DOI:** 10.1111/dme.12392

**Published:** 2014-02-05

**Authors:** F. Bragg, L. Li, M. Smith, Y. Guo, Y. Chen, I. Millwood, Z. Bian, R. Walters, J. Chen, L. Yang, R. Collins, R. Peto, Y. Lu, B. Yu, X. Xie, Y. Lei, G. Luo, Z. Chen, the China Kadoorie Biobank Collaborative Group

**Affiliations:** ^1^ Clinical Trial Service Unit and Epidemiological Studies Unit (CTSU) Nuffield Department of Population Health University of OxfordUK; ^2^ Department of Public Health Beijing UniversityBeijing China; ^3^ Chinese Academy of Medical Sciences Beijing China; ^4^ China National Center for Food Safety Risk Assessment Beijing China; ^5^ Suzhou Centre for Disease Control and Prevention Suzhou China; ^6^ Nangang Centre for Disease Control and Prevention Harbin China; ^7^ Liuyang Centre for Disease Control and Prevention Liuyang China; ^8^ Maiji Centre for Disease Control and Prevention Tianshui China; ^9^ Pengzhou Centre for Disease Control and Prevention Pengzhou China

## Abstract

**Aims:**

To examine the relationship of self‐reported diabetes, and of random blood glucose levels among individuals without known diabetes, with the prevalence of cardiovascular disease in Chinese adults.

**Methods:**

We examined cross‐sectional data from the China Kadoorie Biobank of 0.5 million people aged 30–79 years recruited from 10 diverse regions of China in the period 2004–2008. Logistic regression was used to estimate the odds ratios of prevalent cardiovascular disease associated with self‐reported diabetes, and with measured random blood glucose levels among participants with no history of diabetes, adjusting simultaneously for age, sex, area, education, smoking, alcohol, blood pressure and physical activity.

**Results:**

A total of 3.2% of participants had self‐reported diabetes (men 2.9%; women 3.3%) and 2.8% had screen‐detected diabetes (men 2.6%; women 2.8%), i.e. they had no self‐reported history of diabetes but a blood glucose level suggestive of a diagnosis of diabetes. Compared with individuals without a history of diabetes, the odds ratios associated with self‐reported diabetes were 2.18 (95% CI 2.06–2.30) and 1.88 (95% CI 1.75–2.01) for prevalent ischaemic heart disease and stroke/transient ischaemic attack, respectively. Among participants without self‐reported diabetes there was a positive association between random blood glucose and ischaemic heart disease and stroke/transient ischaemic attack prevalence (*P* for trend <0.0001). Below the diabetic threshold (<11.1 mmol/l) each additional 1 mmol/l of random blood glucose was associated with 4% (95% CI 2–5%) and 5% (95% CI 3–7%) higher odds of prevalent ischaemic heart disease and stroke/transient ischaemic attack, respectively.

**Conclusions:**

In this adult Chinese population, self‐reported diabetes was associated with a doubling of the odds of prevalent cardiovascular disease. Below the threshold for diabetes there was still a modest, positive association between random blood glucose and prevalent cardiovascular disease.

**What's new?**

- Little is known about the role of diabetes as a risk factor for cardiovascular disease in the Chinese population. Below the threshold for diabetes, substantial uncertainty exists about the association of blood glucose levels with cardiovascular disease in Chinese populations and more generally.
- Data from the China Kadoorie Biobank of 0.5 million middle‐aged Chinese adults demonstrated a doubling of the odds of prevalent ischaemic heart disease and stroke/transient ischaemic attack among people with self‐reported diabetes. Among people without prior diabetes, blood glucose levels were positively associated with prevalent cardiovascular disease.
- A comprehensive understanding of the role of both blood glucose levels and diabetes in cardiovascular disease risk is fundamental to effective disease prevention and control.

## Introduction

Cardiovascular disease is the leading cause of morbidity and mortality worldwide 1. Diabetes is an important risk factor for cardiovascular disease; patients with diabetes experience at least a doubling in risk of ischaemic heart disease and stroke compared with those without diabetes 2. There is also evidence to suggest that higher blood glucose levels below the threshold for diabetes may increase the risk of cardiovascular disease 2. In China, the incidence of diabetes has risen rapidly over recent decades 3, but there is limited evidence for the association of diabetes with cardiovascular disease 4, which, in contrast to most Western populations, is characterized in the Chinese population by higher rates of stroke and lower rates of ischaemic heart disease 6. Moreover, uncertainty remains about the association of blood glucose with the risk of cardiovascular disease among the Chinese population without diabetes 7.

To help address these issues, we report cross‐sectional data from the China Kadoorie Biobank of 0.5 million people 8. The objectives were to examine: 1) the relationship between self‐reported, doctor‐diagnosed diabetes and the prevalence of cardiovascular disease; 2) the relevance of age, sex, area, education, smoking status, alcohol consumption, blood pressure, physical activity and adiposity to observed associations; and 3) the association between random blood glucose levels and the prevalence of cardiovascular disease among people without prior diabetes.

## Patients and methods

### Study population

**Figure 1:**
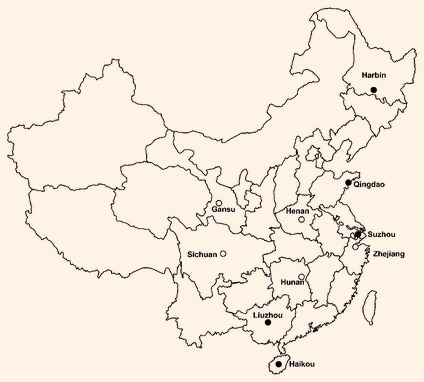
Locations of the China Kadoorie Biobank recruitment centres. Black circles represent urban areas, white circles represent rural areas.

The study design and characteristics of the China Kadoorie Biobank population have been described previously 8. A total of 512 891 men and women aged 30–79 years were recruited between 2004 and 2008 from five urban and five rural areas in China (Fig. 1). The areas were chosen according to local disease patterns, exposure to certain risk factors, population stability, levels of economic development, death and disease registry quality and practical considerations, including local capacity and commitment. In each area, permanent residents of 100–150 administrative units (rural villages or urban residential committees) were identified through official residential records, then invited to participate by letter after extensive publicity campaigns. The population response rate was ~30%.

### Data collection

Data were obtained from participants through interviewer‐administered electronic questionnaires, collating information on demographic and socio‐economic characteristics, lifestyle, personal and family medical history (including a history of doctor‐diagnosed diabetes, ischaemic heart disease —including myocardial infarction and angina — and stroke/transient ischaemic attack) and current medication amongst those reporting ischaemic heart disease, stroke/transient ischaemic attack, hypertension or diabetes. A range of physical measurements were undertaken by trained technicians, including height, weight, hip and waist circumference, bio‐impedance and blood pressure, using calibrated instruments with standard protocols.

A 10‐ml non‐fasting (except in one study area where participants were asked to fast) blood sample was collected from participants into an ethylenediamine tetra‐acetic acid vacutainer (BD Hemogard^TM^, BD, Franklin Lakes, NJ, USA), with the time since the participant last ate recorded. On‐site testing of plasma glucose level was undertaken using the SureStep Plus meter (LifeScan, Milpitas, CA, USA). Participants with glucose levels ≥7.8 mmol/l and <11.1 mmol/l were invited to return for a fasting blood glucose test the next day. Random blood glucose data were unavailable for 8160 participants without self‐reported diabetes (because of a delay in making the on‐site test available in certain regions). Screen‐detected diabetes was defined as no self‐reported diabetes with a blood glucose level ≥7.0 mmol/l and a fasting time >8 h, a blood glucose level ≥11.1 mmol/l and a fasting time <8 h, or a fasting blood glucose level ≥7.0 mmol/l.

Ethical approval for the study was obtained from Oxford University, the China National Centre for Disease Control and Prevention and the 10 study areas’ local Centres for Disease Control and Prevention. All participants provided informed, written consent.

### Statistical analyses

Sex‐specific, age‐ and study area‐adjusted random blood glucose levels in the population without self‐reported diabetes were compared across categories of other variables using general linear models. Prevalence of self‐reported and screen‐detected diabetes were compared across levels of other variables, standardized to 5‐year age groups and study area.

The associations of self‐reported and screen‐detected diabetes, and of random blood glucose level amongst people without self‐reported diabetes, with the prevalence of ischaemic heart disease and stroke/transient ischaemic attack were examined using multivariate logistic regression. Random blood glucose was categorized into six groups (thresholds: 4.8, 5.8, 6.8, 7.8 and 11.1 mmol/l), selected to include the oral glucose tolerance test 2‐h post glucose‐load thresholds for impaired glucose tolerance and diabetes 10. Odds ratios for cardiovascular disease were calculated, adjusting simultaneously for age, study area, education (no formal schooling, primary school, middle school, high school, college/university), smoking (never, occasional, ex‐regular, current regular), and alcohol (never regular, occasional intake, ex‐regular, reduced intake, weekly intake). In separate models, additional adjustments were made for systolic blood pressure (thresholds: 100, 110, 120, 130, 140, 150, 160 and 170 mmHg) and physical activity (thresholds: 10, 20, 30, 40 metabolic equivalent of task h/day), and for waist–hip ratio (thresholds: 0.75, 0.80, 0.85, 0.90 and 0.95).

For random blood glucose analyses, the floating absolute risk method was used to provide estimates of variance across all exposure categories 11. Chi‐squared tests for trend in log odds ratios were conducted and the estimated odds ratios of prevalent ischaemic heart disease and stroke/transient ischaemic attack were examined for departure from linearity by testing the type 3 chi‐square for categorical random blood glucose, in a model that contained random blood glucose as a categorical and a continuous variable. The odds ratios for each additional 1 mmol/l of random blood glucose were estimated in participants with a random blood glucose level <11.1 mmol/l. In participants with neither self‐reported nor screen‐detected diabetes, random blood glucose analyses were repeated, stratified by fasting time (<8 vs ≥8 h). The 8‐h threshold was based on fasting duration guidance 12 and evidence of a threshold in the relationship between random blood glucose and fasting time at 8 h in the China Kadoorie Biobank. Adjusted odds ratios associated with self‐reported diabetes were compared across strata of sex, age, rural/urban, education, smoking status, alcohol consumption, systolic blood pressure, physical activity, adiposity and treatment status (which was additionally adjusted for duration of diabetes diagnosis); chi‐squared tests for trend and heterogeneity were applied to the estimates for each variable 13.

Statistical analyses were conducted using sas version 9.3 (SAS Institute Inc., Cary, North Carolina, USA).

## Results

Overall, 2.9% of men and 3.3% of women reported a history of doctor‐diagnosed diabetes and a further 2.6% of men and 2.8% of women had screen‐detected diabetes. Total diabetes prevalence (combined self‐reported and screen‐detected diabetes) increased with age, and was higher in urban than in rural areas (Table 1). Ex‐regular smokers and drinkers had the highest self‐reported diabetes prevalence compared with other smoking or alcohol categories (Table 1). Screen‐detected diabetes prevalence was highest amongst ex‐regular smokers, but not ex‐regular drinkers. There was a strong inverse association between physical activity and diabetes prevalence. Participants with a family history of diabetes were four times as likely to have doctor‐diagnosed diabetes and approximately twice as likely to have screen‐detected diabetes as those without such a family history (Table 1). Diabetes prevalence was strongly positively associated with systolic blood pressure and adiposity (Table 2).

**Table 1:** Demographic and lifestyle characteristics of China Kadoorie Biobank participants by sex

Characteristic	Men (*n *=* *210 222)				Women (*n *=* *302 669)			
	*n*	Diabetes (%)*		No self‐reported diabetes	*n*	Diabetes (%)*		No self‐reported diabetes
		Self‐reported	Screen‐detected	Mean random blood glucose†^,^‡ (mmol/l)		Self‐reported	Screen‐detected	Mean random blood glucose †^,^‡ (mmol/l)
**Age**§ **(years)**								
30–39	29594	0.7	1.1	5.3	48210	0.3	0.8	5.5
40–49	59230	1.7	2.1	5.6	93519	1.2	1.9	5.8
50–59	63715	3.3	3.0	5.9	93841	3.9	3.5	6.1
60–69	41331	4.9	3.5	6.1	50440	7.2	4.7	6.4
70–79	16352	5.3	4.0	6.2	16659	7.1	4.8	6.4
**Rural/urban**								
Rural	118883	1.6	1.8	5.6	167822	2.4	2.5	5.9
Urban	91339	4.6	3.7	6.0	134847	4.3	3.2	6.0
**Highest level of education**								
No formal schooling	18660	2.8	4.1	5.8	76561	3.4	3.8	6.0
Primary school	70110	2.7	2.9	5.8	95106	3.6	3.2	6.0
Middle school	68172	3.3	2.8	5.8	76741	4.0	2.8	5.9
High school	36727	3.5	2.9	5.8	40800	4.0	2.4	5.8
College or university	16553	4.1	2.7	5.7	13461	3.8	2.0	5.8
**Annual household income (Yuan/year)**								
<2500	6154	2.4	3.7	5.8	9392	2.9	3.9	5.9
2500–4999	13300	2.8	3.1	5.8	21357	3.1	3.0	6.0
5000–9999	35283	2.8	2.3	5.8	59346	3.5	2.7	6.0
10 000–19 999	59558	2.9	2.6	5.8	89455	3.3	3.0	6.0
20 000–34 999	53400	3.1	2.7	5.8	73321	3.4	2.8	6.0
≥35 000	42527	3.8	3.4	5.8	49798	4.1	2.7	6.0
**Smoking**								
Never	30281	3.2	2.6	5.8	287333	3.3	2.8	6.0
Occasional	23628	3.0	2.5	5.8	5531	3.9	3.2	6.0
Ex‐regular	27918	4.1	2.9	5.9	2645	4.9	5.8	6.0
Current regular	128395	2.4	2.6	5.8	7160	4.0	4.3	5.9
**Alcohol**								
Never regular	42764	3.8	2.8	5.8	192435	4.0	3.0	6.0
Occasional	79260	2.7	2.3	5.8	101328	2.4	2.6	5.9
Ex‐regular	7923	7.0	2.6	5.9	1333	8.0	3.3	5.9
Reduced intake	10371	5.5	2.6	5.8	1325	3.4	2.8	6.0
Weekly	69904	1.9	2.8	5.8	6248	1.2	2.2	5.9
**Physical activity (metabolic equivalent of task h/day)**								
<10	23487	4.3	3.7	5.9	15293	5.4	3.7	6.0
10‐19.9	61366	3.7	2.8	5.8	118436	3.9	3.0	6.0
20‐29.9	46417	2.8	2.5	5.8	81247	2.9	2.7	5.9
30‐39.9	36948	1.8	2.5	5.8	45148	2.3	2.6	5.9
≥40	42004	1.5	2.4	5.8	42545	1.5	2.1	5.9
**Family history of diabetes** ¶ ^,^ **								
No	182986	2.4	2.5	5.8	268510	2.7	2.7	5.9
Yes	14225	10.7	4.7	6.1	22347	11.5	4.7	6.2
**Fasting time (h)**								
<8	168500	2.7	2.4	5.9	233913	2.9	2.4	6.1
≥8	41722	5.1	3.9	5.3	68756	6.2	4.1	5.5

*Standardized to the age and study area structure of the study population; ^†^adjusted for age and study area; ^‡^all se values ≤0.1; ^§^adjusted for/standardized to study area only; ^¶^first‐degree relatives; **data missing for 24 823 participants.

**Table 2:** Characteristics of China Kadoorie Biobank participants from physical examination by sex

Characteristic	Men: *n *=* *210 222				Women: *n *=* *302 669			
	*n*	Diabetes (%)*		No self‐reported diabetes	*n*	Diabetes (%)*		No self‐reported diabetes
		Self‐reported	Screen‐detected	Mean random blood glucose†^,^ ‡ (mmol/l)		Self‐reported	Screen‐detected	Mean random blood glucose†^,^‡ (mmol/l)
**Systolic blood pressure**								
<100 mmHg	3940	2.0	1.1	5.5	13595	1.3	1.2	5.7
100–119 mmHg	50915	2.0	1.5	5.6	94810	2.0	1.6	5.8
120–139 mmHg	90876	2.8	2.4	5.8	110743	3.0	2.7	6.0
140–159 mmHg	43526	3.5	3.7	6.0	53187	4.6	4.1	6.2
≥160 mmHg	20965	4.1	4.8	6.1	30334	5.5	5.3	6.2
**BMI** §								
<18.5 kg/m^2^	9426	1.8	1.7	5.7	12947	1.7	1.7	5.7
18.5 to <22.5 kg/m^2^	77017	2.0	1.7	5.6	99120	2.7	1.7	5.8
22.5 to <25 kg/m^2^	58581	3.1	2.5	5.8	86868	3.4	2.6	5.9
25 to <30 kg/m^2^	58975	3.9	3.8	6.0	88990	3.9	3.9	6.1
≥30 kg/m^2^	6222	5.5	6.4	6.4	14743	4.9	5.9	6.5
**Waist circumference**								
<70 cm	21361	1.2	1.3	5.6	50518	1.4	1.2	5.7
70–79.9 cm	70042	1.8	1.5	5.6	117308	2.6	1.9	5.8
80–89.9 cm	72494	3.2	2.6	5.8	94766	3.8	3.4	6.1
90–99.9 cm	38021	4.3	4.3	6.1	33317	5.0	5.3	6.4
≥100 cm	8304	5.9	7.0	6.5	6760	7.0	7.8	6.8
**Hip circumference**								
<84 cm	33453	1.9	2.2	5.7	40403	2.7	2.2	5.8
84–87.9 cm	41832	2.3	1.9	5.7	58436	3.5	2.5	5.9
88–91.9 cm	48447	2.9	2.4	5.8	73849	3.4	2.6	5.9
92–95.9 cm	40464	3.4	2.9	5.8	61553	3.4	3.0	6.0
≥96 cm	46026	3.8	3.9	6.0	68428	3.6	3.8	6.1
**Waist**–**hip ratio**								
<0.75	987	1.1	1.8	5.5	9793	1.0	1.0	5.5
0.75–0.80	8073	1.1	1.2	5.5	36890	1.2	1.1	5.6
0.80–0.85	29810	1.2	1.2	5.5	72148	1.9	1.6	5.8
0.85–0.90	55847	2.1	1.7	5.6	83560	3.0	2.5	5.9
0.90–0.95	60599	3.0	2.5	5.8	60535	4.3	3.9	6.2
≥0.95	54906	4.8	4.6	6.2	39743	6.5	6.4	6.6
**Percentage body fat** ¶								
<15	27510	2.1	1.4	5.7	1125	2.0	2.1	5.9
15–24.9	117804	2.8	2.0	5.7	45652	2.3	1.4	5.8
25–34.9	59762	3.7	4.1	6.0	157011	3.3	2.3	5.9
≥35	5022	4.1	6.9	6.3	98764	3.8	4.3	6.1

*Standardized to the age and study area structure of the study population; ^†^adjusted for age and study area; ^‡^all se values ≤0.1; ^§^data missing for two participants; ^¶^ data missing for 241 participants.

Mean (se) age‐ and area‐adjusted random blood glucose was 5.8 (<0.1) mmol/l in men and 6.0 (<0.1) mmol/l in women without self‐reported diabetes. Random blood glucose varied little by socio‐economic or lifestyle factors, but increased with increasing age (Table 1), systolic blood pressure and adiposity (Table 2).

**Figure 2:**
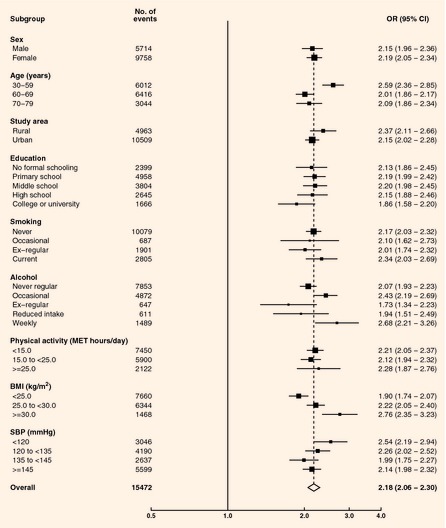
Odds ratios (ORs) for prevalent ischaemic heart disease by self‐reported diabetes status. Adjusted for age, sex, study area, education, smoking, alcohol, systolic blood pressure, physical activity. Closed squares represent the OR with area inversely proportional to the variance. Horizontal lines represent the corresponding 95% CIs. The dotted line indicates the overall OR. The open diamond represents the overall OR and its 95% CI. BMI, body mass index; MET, metabolic equivalent of task; SBP, systolic blood pressure.

**Figure 3:**
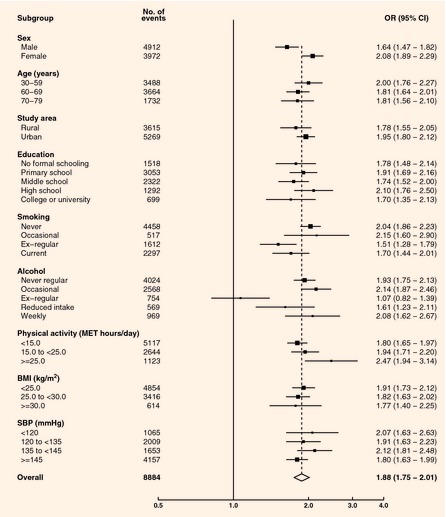
Odds ratios (ORs) for prevalent stroke/transient ischaemic attack by self‐reported diabetes status. Adjusted for age, sex, study area, education, smoking, alcohol, systolic blood pressure, physical activity. Closed squares represent the OR with area inversely proportional to the variance. Horizontal lines represent the corresponding 95% CIs. The dotted line indicates the overall OR. The open diamond represents the overall OR and its 95% CI. BMI, body mass index; MET, metabolic equivalent of task; SBP, systolic blood pressure.

A total of 2.7% of men and 3.2% of women reported a history of doctor‐diagnosed ischaemic heart disease, higher than for stroke/transient ischaemic attack (men 2.3%; women 1.3%). The prevalence of ischaemic heart disease and of stroke/transient ischaemic attack increased with increasing age, and were higher in urban than in rural areas (ischaemic heart disease: 4.6 vs 1.7%; stroke/transient ischaemic attack: 2.3 vs 1.3%). After adjusting for age, area, education, smoking, alcohol, systolic blood pressure and physical activity, individuals with self‐reported diabetes were more than twice as likely to have ischaemic heart disease (men: odds ratio 2.15, 95% CI 1.96–2.36; women: odds ratio 2.19, 95% CI 2.05–2.34) than those without (Table 3). Similar findings were observed for stroke/transient ischaemic attack, with a somewhat greater odds ratio in women (odds ratio 2.08, 95% CI 1.89–2.29) than in men [odds ratio 1.64, 95% CI 1.48–1.82 (*P* for interaction=0.0006)]. Adjusted odds ratios for prevalent ischaemic heart disease (IHD) with self‐reported diabetes were similar across the strata of other risk factors with the exception of age and BMI, with a higher odds in participants aged 30–59 years (*P *=* *0.001 for trend), and increasing odds with increasing BMI [*P *<* *0.001 for trend (Fig. 2)]. The odds ratios for prevalent stroke/transient ischaemic attack increased with increasing physical activity levels (*P *=* *0.02 for trend) but varied little by other risk factors (Fig. 3). Further adjustment for waist–hip ratio moderately attenuated both associations.

**Table 3:** Odds ratios for prevalent cardiovascular diseases by self‐reported diabetes status in men and women

	Self‐reported diabetes	No self‐reported diabetes	Age‐adjusted		Model A		Model B		Model C	
	Events/ Participants	Events/ Participants	OR	95%CI	OR	95%CI	OR	95%CI	OR	95%CI
**Ischaemic heart disease**										
Men	683/ 6124	5031/ 204098	3.37	(3.09–3.68)	2.25	(2.06–2.47)	2.15	(1.96–2.36)	2.00	(1.82–2.19)
Women	1370/ 10038	8388/ 292631	3.03	(2.85–3.23)	2.31	(2.16–2.47)	2.19	(2.05–2.34)	2.04	(1.90–2.18)
**Stroke/transient ischaemic attack**										
Men	505/ 6124	4407/ 204098	2.76	(2.51–3.05)	1.85	(1.67–2.06)	1.64	(1.48–1.82)	1.55	(1.39–1.72)
Women	602/ 10038	3370/ 292631	3.06	(2.80–3.36)	2.48	(2.25–2.72)	2.08	(1.89–2.29)	1.93	(1.75–2.12)

Model A: adjusted for age, study area, education, smoking, alcohol; Model B: additionally adjusted for systolic blood pressure and physical activity; Model C: additionally adjusted for waist–hip ratio. OR, odds ratio.

Of the 13 678 (84.6%) participants with self‐reported diabetes with data available on hypoglycaemic medication use, 76.9% reported taking such medication (84.7% oral hypoglycaemics; 18.9% insulin). The odds ratio for ischaemic heart disease, after adjustment for other cardiovascular disease risk factors, was significantly higher in participants reporting hypoglycaemic medication use than in those not using such medication (odds ratio 2.25 vs 1.69; *P* for heterogeneity <0.001; Fig. S1). This difference was minimally attenuated after adjustment for duration of diabetes diagnosis. The risk of prevalent stroke/transient ischaemic attack was non‐significantly elevated in those who reported use of hypoglycaemic medications (*P* for heterogeneity 0.1; Fig. S1).

After adjustment for age, area, education, smoking, alcohol, systolic blood pressure and physical activity, there was no significant difference in the odds of prevalent ischaemic heart disease (men: odds ratio 0.92, 95% CI 0.80–1.07; women: odds ratio 0.91, 95% CI 0.82–1.01) or stroke/transient ischaemic attack (men: odds ratio 0.95, 95% CI 0.81–1.10; women: odds ratio 1.15, 95% CI 1.00–1.32) between individuals with screen‐detected diabetes and those without any diabetes.

**Figure 4:**
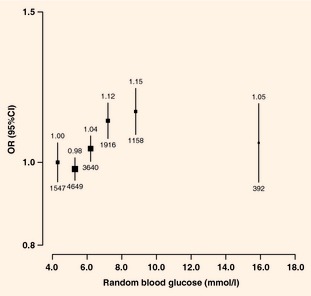
Odds ratios (ORs) for prevalent ischaemic heart disease by random blood glucose levels in participants without self‐reported diabetes. Adjusted for age, sex, study area, education, smoking, alcohol, systolic blood pressure and physical activity. ORs are plotted against mean random blood glucose level in each group (<4.8/4.8–5.7/5.8–6.7/6.8–7.7/7.8–11.0/≥11.1 mmol/l). Squares represent the OR with area inversely proportional to the variance. Vertical lines represent the corresponding 95% CIs. Numbers above the CIs are the ORs and numbers below the CIs are represent participants with self‐reported ischaemic heart disease.

**Figure 5:**
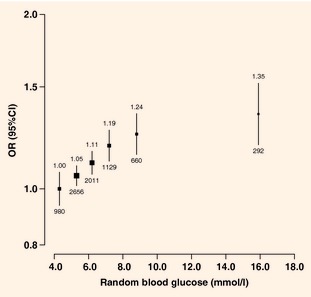
Odds ratios (ORs) for prevalent stroke/transient ischaemic attack by random blood glucose levels in participants without self‐reported diabetes. Adjusted for age, sex, study area, education, smoking, alcohol, systolic blood pressure and physical activity. ORs are plotted against mean random blood glucose level in each group (<4.8/4.8–5.7/5.8–6.7/6.8–7.7/7.8–11.0/≥11.1 mmol/l). Squares represent the OR with area inversely proportional to the variance. Vertical lines represent the corresponding 95% CIs. Numbers above the CIs are the ORs and numbers below the CIs represent participants with self‐reported stroke/transient ischaemic attack.

Among participants without self‐reported diabetes, there was a positive association between random blood glucose levels and ischaemic heart disease prevalence, with adjusted odds ratios of 1.00, 0.99, 1.05, 1.14, 1.18 and 1.13 at random blood glucose levels of <4.8 (reference), 4.8–5.7, 5.8–6.7, 6.8–7.7, 7.8–11.0 and ≥11.1 mmol/l, respectively (*P* for trend <0.0001). After further adjustment for systolic blood pressure and physical activity, the odds ratios were attenuated, but a significant trend remained (*P* for trend <0.0001; Fig. 4). Below 11.1 mmol/l there was no significant deviation from log‐linearity of the association, with each additional 1 mmol/l of random blood glucose associated with 4% (95% CI 2–5%) higher odds of prevalent ischaemic heart disease. The prevalence of stroke/transient ischaemic attack increased with higher random blood glucose levels, with adjusted odds ratios of 1.00, 1.06, 1.14, 1.23, 1.35 and 1.64 at random blood glucose levels of <4.8 (reference), 4.8–5.7, 5.8–6.7, 6.8–7.7, 7.8–11.0 and ≥11.1 mmol/l, respectively (*P* for trend <0.0001). Additional adjustment for systolic blood pressure and physical activity attenuated the associations, but the trend remained significant and did not deviate significantly from log‐linearity (*P* for trend <0.0001; Fig. 5). Below 11.1 mmol/l, each additional 1 mmol/l of random blood glucose was associated with 5% (95% CI 3–7%) greater odds of prevalent stroke/transient ischaemic attack. Additional adjustment for waist–hip ratio attenuated the associations of random blood glucose with ischaemic heart disease (odds ratios of 0.96, 1.00, 1.05, 1.06 and 0.93 at random blood glucose levels of 4.8–5.7, 5.8–6.7, 6.8–7.7, 7.8–11.0 and ≥11.1 mmol/l compared with <4.8 mmol/l; *P* for trend 0.046) and stroke/transient ischaemic attack (odds ratios of 1.04, 1.07, 1.12, 1.17 and 1.21; *P* for trend <0.0001). There was no significant difference in the association of fasting and non‐fasting blood glucose with the prevalence of ischaemic heart disease or stroke/transient ischaemic attack (Fig. S2).

## Discussion

The present study is the largest cross‐sectional study on the associations of diabetes, and of blood glucose levels among adults without diabetes, with cardiovascular disease in a Chinese population. The study showed an independent doubling of the odds of prevalent ischaemic heart disease and stroke/transient ischaemic attack associated with selfreported diabetes. Amongst individuals without diabetes diagnoses there was an apparent positive association of random blood glucose levels with both these conditions.

A few Chinese studies have previously reported the positive association of diabetes with prevalent IHD. The China National Diabetes and Metabolic Disorders Study, a nationally representative survey of >45 000 adults aged ≥20 years, found that diabetes (based on self‐report and oral glucose tolerance test) was associated with an odds ratio of 2.44 for ischaemic heart disease 4. In another study of almost 60 000 adults aged >40 years in rural Beijing, self‐reported diabetes was associated with an odds ratio of 2.51 for ischaemic heart disease 5. Our point estimates are lower, but not inconsistent with these studies; the differences may reflect different disease definitions 4 or variable adjustment for confounders 5. Our findings are more consistent with prospective studies, which have tended to adjust for confounders more comprehensively. In a large, individual participant data meta‐analysis of almost 700 000 people from predominantly Western populations, diabetes (defined variably by self‐report, medication use or fasting blood glucose levels) was associated with a doubling of risk for ischaemic heart disease in a fully adjusted model 14. In the Asia Pacific Cohort Studies Collaboration individual participant data meta‐analysis involving 161 214 participants, self‐reported diabetes was associated with a 73% excess risk of ischaemic heart disease after adjustment for sex, study and age, which persisted after further adjustment for systolic blood pressure, cholesterol, obesity and smoking 15.

Glycaemic thresholds for diabetes are based on elevated microvascular disease risk 10 and may be less relevant to macrovascular disease 2. One cross‐sectional US study of 2500 individuals without known diabetes but with hypertension or hyperlipidaemia, found a significantly elevated independent risk of self‐reported ischaemic heart disease (*n *=* *1274) at all fasting blood glucose levels > 4.8 mmol/l compared with < 4.4 mmol/l 16. To our knowledge, no studies in mainland China have published data on the association of blood glucose with ischaemic heart disease or stroke risk amongst individuals without diabetes. One prospective study involving 16 500 individuals without diabetes in Taiwan showed no significant association of composite cardiovascular disease deaths with post‐challenge (relative risk 1.61, 95% CI 0.86–2.99) or fasting (relative risk 0.84, 95% CI 0.47–1.51) blood glucose, comparing the highest and lowest quintiles 7. Several prospective studies have found an increased risk of ischaemic heart disease only at or above glycaemic thresholds for diabetes, impaired glucose tolerance or impaired fasting glucose 14. An individual participant data meta‐analysis, including > 250 000 participants without known diabetes and ~13 000 ischaemic heart disease events, found a significantly elevated risk only at fasting blood glucose levels > 6.1 mmol/l 14. Other studies, however, have reported a significant positive association within the ‘normoglycaemic’ range 19; the Asia Pacific Cohort Studies Collaboration found a log‐linear association with ischaemic heart disease extending down to a fasting blood glucose level of 4.9 mmol/l 19. These apparent inconsistencies may reflect differences in populations studied, sample size, glycaemic measures, adjustment for confounders or intra‐individual variation in blood glucose levels and reverse causality. Few studies have examined the relationship with random blood glucose but, in contrast to the findings presented, a published data meta‐analysis including almost 10 000 participants and ~300 ischaemic heart disease events showed no significant association of random blood glucose with fatal ischaemic heart disease (hazard ratio 1.02), although the level of adjustment is unclear 20. Our main results were based on random blood glucose, but there was no material difference in associations after stratifying by fasting time, although the statistical power was limited by small event numbers in some categories. Our main analyses did not adjust for adiposity as it is causally related to diabetes and increased random blood glucose, as confirmed by significant attenuation of associations between random blood glucose and prevalent cardiovascular disease after adjustment for adiposity.

Evidence on the association of diabetes with stroke in Chinese populations is limited. In the cross‐sectional rural Beijing study, self‐reported diabetes was associated with a twofold greater prevalence of stroke 5. Several large prospective studies of non‐Chinese populations 21, including meta‐analyses 15, have shown an approximately one‐and‐a‐half to threefold greater risk of total stroke associated with diabetes 15, similar to our study findings. We found significantly higher odds of prevalent stroke/transient ischaemic attack, but not ischaemic heart disease, amongst women than men. A more adverse diabetes‐associated risk profile for cardiovascular disease in women and treatment differences favouring men have been suggested as possible explanations for the greater risk in women 24.

Associations between blood glucose levels and stroke have been examined primarily in non‐Chinese populations 18. Two large studies showed positive associations of fasting blood glucose with stroke risk within the ‘normoglycaemic’ range 18. A study of >15 000 adults in Scotland estimated hazard ratios of 1.07 and 1.12 for total stroke in men and women, respectively, per 1 sd higher random blood glucose 27. The stronger relationship of random blood glucose with stroke/transient ischaemic attack than with ischaemic heart disease in the China Kadoorie Biobank was also observed for fasting blood glucose in a study of Korean men 18, but this contrasts with studies in many Western populations demonstrating no difference 27 or possibly a stronger association with ischaemic heart disease than with stroke 28. The role of small vessel pathology in cerebrovascular disease, thought to be particularly prominent in Chinese populations 29, could explain the stronger association with stroke/transient ischaemic attack.

Previous studies have shown nonsignificantly lower risks of ischaemic heart disease or stroke in screen‐detected diabetes (or amongst individuals with a comparable glycaemic status) than in self‐reported diabetes 14. The markedly lower odds of cardiovascular disease associated with screen‐detected than with self‐reported diabetes in the present study may reflect shorter disease duration or less severe glycaemic aberrations in screen‐detected diabetes, selective diagnosis of diabetes amongst individuals with cardiovascular disease or a greater proportion of false‐positive diabetes diagnoses in the screen‐detected group.

The size and diversity of the China Kadoorie Biobank data enable reliable estimates of the relationships of diabetes and blood glucose levels with prevalent cardiovascular disease. Although not designed to be nationally representative, the estimated diabetes prevalence of 5.9% is reasonably consistent with estimates from nationally representative surveys in China 30. In the China National Diabetes and Metabolic Disorder Study, self‐reported ischaemic heart disease and stroke prevalences were <1% 4. In contrast, the rural Beijing study reported ischaemic heart disease and stroke prevalences of 5.6% and 3.7%, respectively 5, higher than estimates in the present study. Differences between studies probably reflect differences in disease definitions, sampling schemes and populations or temporal trends 3.

Self‐reporting of diabetes is prone to error but in a 2008 resurvey of ~20 000 randomly selected China Kadoorie Biobank participants, ~90% of participants who reported diabetes at baseline again reported a history of diabetes. Arguably, more robust approaches to glycaemic status assessment exist but are less feasible in large population‐based studies. The cross‐sectional design is susceptible to bias from reverse causality. Since diabetes has been associated with a higher fatal than non‐fatal risk of cardiovascular disease 14 and higher cardiovascular disease mortality rates 32, the use of non‐fatal outcomes — and potentially less severe forms — could underestimate associations. Furthermore, a known diagnosis of diabetes could bias self‐reporting of cardiovascular disease. Our inability to adjust for the effects of lipids may have produced residual confounding.

Our analyses provide clear evidence of an independently elevated prevalence of ischaemic heart disease and stroke/transient ischaemic attack associated with self‐reported diabetes. They also provide supportive evidence of associations of random blood glucose levels below the diabetic threshold with prevalent stroke/transient ischaemic attack and possibly with ischaemic heart disease, although their independence is unclear. Multiple pathophysiological explanations for associations of diabetes and blood glucose levels with cardiovascular disease exist 2, and a sound understanding of the role of both exposures in determining ischaemic heart disease and stroke risk is fundamental to effective prevention and control of cardiovascular disease in Chinese and other populations. Continuing follow‐up for incident ischaemic heart disease and stroke among the China Kadoorie Biobank participants will provide large‐scale prospective evidence about these relationships.

## Funding sources

The China Kadoorie Biobank baseline survey and first re‐survey in China were supported by the Kadoorie Charitable Foundation in Hong Kong; follow‐up of the project during 2009–2014 is supported by the Wellcome Trust in the UK (grant 088158/Z/09/Z); the Clinical Trial Service Unit and Epidemiological Studies Unit at Oxford University also receives core funding for the study from the UK Medical Research Council, the British Heart Foundation and Cancer Research UK.

## Competing interests

None declared.

## Members of the China Kadoorie Biobank collaborative group

(a) International Steering Committee: Liming Li, Zhengming Chen, Junshi Chen, Rory Collins, Fan Wu (ex‐member), Richard Peto.

(b) Study coordinating centres: International Co‐ordinating Centre, Oxford: Zhengming Chen, Garry Lancaster, Xiaoming Yang, Alex Williams, Margaret Smith, Ling Yang, Yumei Chang, Iona Millwood, Yiping Chen, Qiuli Zhang, Sarah Lewington, Gary Whitlock. National Co‐ordinating Centre, Beijing: Yu Guo, Guoqing Zhao, Zheng Bian, Can Hou, Yunlong Tan. Regional Co‐ordinating Centres, 10 areas in China:

*Qingdao*

Qingdao Centre for Disease Control: Zengchang Pang, Shanpeng Li, Shaojie Wang,

Licang Centre for Disease Control: Silu lv.

*Heilongjiang*

Provincial Centre for Disease Control: Zhonghou Zhao, Shumei Liu, Zhigang Pang

Nangang Centre for Disease Control: Liqiu Yang, Hui He, Bo Yu.

*Hainan*

Provincial Centre for Disease Control: Shanqing Wang, Hongmei Wang

Meilan Centre for Disease Control: Chunxing Chen, Xiangyang Zheng.

*Jiangsu*

Provincial Centre for Disease Control: Xiaoshu Hu, Minghao Zhou, Ming Wu, Ran Tao,

Suzhou Centre for Disease Control: Yeyuan Wang, Yihe Hu, Liangcai Ma

Wuzhong Centre for Disease Control: Renxian Zhou.

*Guangxi*

Provincial Centre for Disease Control: Zhenzhu Tang, Naying Chen, Ying Huang

Liuzhou Centre for Disease Control: Mingqiang Li, Zhigao Gan, Jinhuai Meng, Jingxin Qin.

*Sichuan*

Provincial Centre for Disease Control: Xianping Wu, Ningmei Zhang

Pengzhou Centre for Disease Control: Guojin Luo, Xiangsan Que, Xiaofang Chen.

*Gansu*

Provincial Centre for Disease Control: Pengfei Ge, Xiaolan Ren, Caixia Dong

Maiji Centre for Disease Control: Hui Zhang, Enke Mao, Zhongxiao Li.

*Henan*

Provincial Centre for Disease Control: Gang Zhou, Shixian Feng

Huixian Centre for Disease Control: Yulian Gao, Tianyou He, Li Jiang, Huarong Sun.

*Zhejiang*

Provincial Centre for Disease Control: Min Yu, Danting Su, Feng Lu

Tongxiang Centre for Disease Control: Yijian Qian, Kunxiang Shi, Yabin Han, Lingli Chen.

*Hunan*

Provincial Centre for Disease Control: Guangchun Li, Huilin Liu, LI Yin

Liuyang Centre for Disease Control: Youping Xiong, Zhongwen Tan, Weifang Jia.

## Supplementary Material

**Figure S1** Odds ratios (OR) for prevalent CVD by hypoglycaemic medication treatment status in selfreported diabetes.

**Figure S2** Odds ratios (OR) for (a) prevalent IHD and (b) prevalent stroke/TIA by random blood glucose levels in participants without diabetes (self‐reported or screen‐detected) by fasting time.
